# Intranasal Air Suction Approach for Management of Acute Migraine Headache: An International Perspective

**DOI:** 10.1192/j.eurpsy.2026.10931

**Published:** 2026-07-31

**Authors:** J. Jeyapalan, M. Nissila, S. Sihvonen

**Affiliations:** 1 School of Health and Social Studies, Jyväskylä University of Applied Sciences, Jyväskylä; 2 Department of Psychiatry, University of Turku; 3 clinical Research and Biobank, Suomen Terveystalo, Turku, Finland

## Abstract

**Introduction:**

Migraine is a disabling neurological disorder affecting over one billion people worldwide. Despite advances in treatment, the precise mechanisms remain unclear. The Sinus Hypoxic Nitric Oxide Theory (SHNOT) suggests that excessive sinus nitric oxide (sNO), generated under hypoxic conditions, plays a central role in migraine pathophysiology.

**Objectives:**

This study aimed to evaluate and compare two nasal-based interventions—intranasal drug administration (INDAA) and paranasal air suction approach (PNASA)—for acute migraine management. Specific objectives were to:

1. Assess their efficacy in rapid symptom relief,
2. Compare safety and side-effect profiles,
3. Examine the mechanistic role of sNO and sCO,
4. Validate SHNOT as a theoretical framework.

**Methods:**

An integrative review was conducted. Literature searches in PubMed, Google Scholar, and Cochrane identified 312 records. After screening, 11 studies met the inclusion criteria. Thematic analysis was applied across randomised trials, case series, meta-analyses, and gas analysis studies.

**Results:**

INDAA agents (zolmitriptan, lidocaine, hydroxocobalamin, oxymetazoline) showed variable efficacy, often accompanied by local irritation or systemic effects. In contrast, PNASA studies consistently demonstrated rapid migraine relief within 1–2 minutes, with no adverse effects reported (Figure 1).

**Table 1.** summarises key outcomes Table 1. Long description.

Intervention	N (participants)	Onset of Relief	Adverse Effects
Zolmitriptan (5 mg nasal spray)	798	≤2 h	Mild systemic effects
Intranasal Lidocaine	134 (meta-analysis)	≤5 min	Local irritation (~49%)
Hydroxocobalamin (1 mg intranasal)	20	Prophylactic (3 mo)	Well tolerated
Oxymetazoline (0.1%)	108	≤15 min	Potential rebound congestion
PNASA (air suction)	184	1–2 min	None observed

**Image 1:**
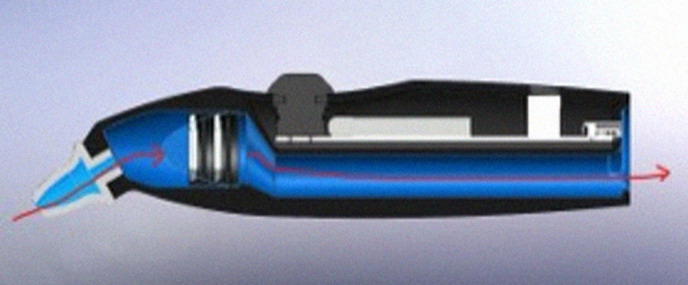

**Conclusions:**

Paranasal air suction represents a safe, non-invasive, and cost-effective acute migraine treatment. Findings strongly support SHNOT, positioning sNO as a key causative molecule. PNASA shows superior efficacy and tolerability compared to INDAA, highlighting its potential as a first-line intervention in diverse healthcare settings.

**Disclosure of Interest:**

None Declared

## Long descriptions

### Table 1. Long description

The table consists of four columns: Intervention, N (participants), Onset of Relief, and Adverse Effects.

* Row 1: Zolmitriptan (5 mg nasal spray); 798 participants; Onset of Relief is less than or equal to 2 h; Adverse Effects are mild systemic effects.

* Row 2: Intranasal Lidocaine; 134 participants (meta-analysis); Onset of Relief is less than or equal to 5 min; Adverse Effects include local irritation in approximately 49% of cases.

* Row 3: Hydroxocobalamin (1 mg intranasal); 20 participants; Onset of Relief is prophylactic (3 mo); Adverse Effects are noted as well tolerated.

* Row 4: Oxymetazoline (0.1%); 108 participants; Onset of Relief is less than or equal to 15 min; Adverse Effects include potential rebound congestion.

* Row 5: PNASA (air suction); 184 participants; Onset of Relief is 1 to 2 min; No adverse effects were observed.

Navigate back to Table 1..

